# Technology Development as a Normative Practice: A Meaning-Based Approach to Learning About Values in Engineering—Damming as a Case Study

**DOI:** 10.1007/s11948-017-9999-7

**Published:** 2017-11-10

**Authors:** Mahdi G. Nia, Mehdi F. Harandi, Marc J. de Vries

**Affiliations:** 1 Delft University of Technology, Delft, The Netherlands; 2 University of Tehran, Tehran, Iran

**Keywords:** Normative practice, Technology development, Normativity, Dooyeweerd’s ontological account, Normative constitutive rules, Normative regulative rules

## Abstract

Engineering, as a complex and multidimensional practice of technology development, has long been a source of ethical concerns. These concerns have been approached from various perspectives. There are ongoing debates in the literature of the philosophy of engineering/technology about how to organize an optimized view of the values entailed in technology development processes. However, these debates deliver little in the way of a concrete rationale or framework that could comprehensively describe different types of engineering values and their multi-aspect interrelations in real engineering practices. Approaching engineering values from a meaning-based perspective, as in this paper, can be a reliable method of tackling such a controversial problem. This paper therefore proposes that technology development be considered a systemic normative practice and attempts to provide a comprehensive view of various built-in values, their different origins and features, and a way of prioritizing them in real engineering processes. Studying two cases of the Zayandeh Rood Dam and the Abbasi Dam will lead to practical insights into how to understand norms in technology development and incorporate them into engineering practice.

## Introduction

The practice of engineering has always been a source of ethical concerns. As a multidimensional practice involving technology development, it has complex interrelations with many other activities, and ethical considerations of its potential impact have been approached from various perspectives.

Accordingly, the literature of the philosophy of engineering/technology has included much debate over the establishment of concrete rationale(s) or framework(s) that could comprehensively describe ‘engineering values’ (see, e.g., Clift 2011; Didier 2009; Doorn and Fahlquist 2010; Dupuy 2009; Keulartz 2009; Kroes et al. 2009; Mitcham and Briggle 2009; Mitcham and Waelbers 2009; Pitt 2011; Van de Poel 2009; Van de Poel and Verbeek 2006; Stirling 2011; Swierstra and Jelsma 2006; Waelbers 2009). Such attempts become even more controversial when faced with differing perceptions of the complicated, multifaceted nature of engineering practice (Clift 2011; Didier 2009; Doorn and Fahlquist 2010; Keulartz 2009; Kroes et al. 2009; Van de Poel 2009; Waelbers 2009), so that the idea of organizing an optimized, overarching view of the values entailed in technology development processes seems idealistic, inaccessible, and perhaps nothing more than a blind alley, in the opinion of some scholars (see, e.g., Didier 2009; Keulartz 2009; Kroes et al. 2009; Pitt 2011; Simon 1973, 1976, 1996; Van de Poel 2009). In view of this, the main question to be dealt with in this article is how to tackle such difficulties and contribute to organizing those values in an overarching view, based on a concrete, practical foundation.

We would like to embark on the discussion by stating that the field of ‘engineering ethics’ is, in fact, not old; it can be traced back to the 1970s (Doorn and Fahlquist 2010). However, this area, typically perceived as a field of *applied ethics*, has undergone various critiques and modifications, in the sense of being tailored to actual practices (see, e.g., Clift 2011; Doorn and Fahlquist 2010; Lynch and Kline 2000; Mitcham and Briggle 2009; Mitcham and Waelbers 2009; Peterson 2009; Van de Poel 2009; Stirling 2011; Swierstra and Jelsma 2006). One of the most common and earliest concerns as to the applicability of this applied ethics is related to its traditional focus on matters such as ‘individual responsibilities in technological failures or disasters’ (which mainly concentrates on the role of individuals rather than collectives and professions as a whole in ethical considerations), ‘blameworthiness orientation’ [based upon “seeking cause of failures and assessing blame … putting constraints in place to correct for whatever actions or inactions occurred leading to the problem” (Pitt 2011, p. 126)], and ‘so-called whistle-blowing policies’ (as to how and when individual engineers should blow the whistle in the case of expecting certain disasters) (Didier 2009; Doorn and Fahlquist 2010; Durbin 2008; Lynch and Kline 2000; Pritchard 2001; Pritchard and Holtzapple 1997; Swierstra and Jelsma 2006; Vanderburg 2000). This traditional approach, however, concentrates mostly on ‘wrongdoings’ and less on ‘the positive standards’ that responsible engineers ought to follow; furthermore, it ignores the matter of ‘collective responsibility’ of various actors—the problem of *many hands*, which makes it difficult to find an individual responsible for some failures in the case of complex collective actions (Doorn and Fahlquist 2010)—in technological practices. Another drawback to this traditional approach is its lack of attention to *institutional ethics* (which considers the role of institutions rather than individuals behind many significant ethical decisions), the fact that could lead engineers toward the ‘trap of (hidden) duality’ placing them against upper-level policy makers, particularly managers, whereas a main requirement of engineers is loyalty to the decision hierarchy of their organization (Boudon 1979; Giddens 1984; Swierstra and Jelsma 2006).

The other parallel or subsequent approaches of applied ethics, too, have received considerable critiques by the philosophers. For instance, ‘Codes of ethics’ (as certain value-based standards to be followed in engineering processes) are considered to present significant difficulties in delineating the exact responsibilities of engineers in the face of real organizational issues and value conflicts (Clift 2011; Mitcham and Briggle 2009). Such codes seem that have their roots more in engineers’ reflections on their practice than in those of the philosophers of engineering or technology (Mitcham and Briggle 2009) and, consequently, do not have a stable base to view the state of values in different situations and explain them appropriately (Clift 2011; Didier 2009; Mitcham and Briggle 2009).

In the same vein, ‘(instrumental) rationality’ (which attempts to present a more concrete sense, as compared to its predecessors, of values conflicts and different approaches to tackling them in rational procedures), is subject to significant concerns, particularly as to its limited assessment-based power in facing the multi-criteria practices of various conflicts or so-called *incommensurable* issues^1^ (Kroes et al. 2009; Simon 1973; Van de Poel 2009). Likewise, there are other more or less similar arguments questioning the applicability of the approaches of ‘statement of ethical principles for engineering’, ‘precautionary principle’, ‘efficiency’, and so forth (Clift 2011; Mitcham and Briggle 2009; O’Neill 2011; Stirling 2009, 2011; Van de Poel 2009).

That said, the current philosophical reflections on ethics of engineering/technology could scarcely address a comprehensive solution to the above problems, and the issue of reaching an overarching description, applicable in real practice, still remains. One can see, for instance, that Van de Poel’s (2009) respectful ideas as to the necessity of considering the matter of ‘diversity’ and ‘genre-specificity’ of ethical issues are still proposed in the line of the above-mentioned *rationalistic* approach; the worthy concept of ‘value-sensitive design’, proposed by Van der Hoven and Manders-Huits (2009), concentrates on a *preventive* approach tackling different values as much as possible in the design phase; Doorn and Fahlquist’s (2010) innovative suggestion endeavours to enhance the state of ethical considerations through entering engineering ethicists into the research teams of technology development; Swierstra and Jelsma (2006) propose integrating engineering ethicists in the structural levels of decision making in companies; Lynch and Kline’s (2000) prudent discussions lead to highlighting the necessity of incorporating ethics-oriented knowledge and skills in educational plans for engineering, etc.—however, the matter of providing a concrete rationale able to prioritize values and tackle the conflicts within actual practices of engineering is still a substantial issue.

In some recent discussions, this problem has been considered to have its roots mainly in the insufficient attention to the matter of *normativity* within a great part of customary approaches of ethical reflections on engineering and technology; the approaches, although leading to rich and cumulative insights, remain largely ‘theoretical’ and ‘descriptive’ in character and still need to be enriched more in the sense of ‘practical’ and ‘prescriptive’ orientations while, concurrently, taking the matter of normativity into serious account (Borgmann 2006; van Burken and de Vries 2012; Harandi et al. 2014; Mitcham and Waelbers 2009; Jochemsen 2006, 2013, 2015). Accordingly, one can raise two relevant questions:

- Given the complex and multifaceted nature of most technology development practices, how can we explore their nature and underpin a well-organized and applicable account that can prioritize different values and the raised conflicts within actual engineering practices?
- And, if such an applicable account is to be normative, what view can provide a concrete rationale for describing such normativity?

These are the main questions this study aims to address.

This article is based upon a foundation of two correlative perspectives. The first is the necessity of approaching technology development practices—and their complexities—through a ‘systemic view’. This will lead to the worth of underpinning a sound approach able to deliberate the nature and different aspects of such systems (“Technology Development as a Systemic Multi-aspect Practice” section). It is worth mentioning that speaking about ‘technology development’ in this paper embraces a holistic perspective on technology—considering it from the four different lenses of technology as *artefact*, *knowledge*, *activity*, and *volition* (human/social will), as proposed in Mitcham’s (1994) account .

The second is the significance of sidestepping the customary view of modernity in seeing values and ethical issues as *external subjective additives* to technological practices, the view dominant within most current applied ethics (Doorn and Fahlquist 2010; Glas 2012; Jochemsen 2006; Pitt 2011). This consideration suggests a normativity-based orientation which realises values as *internal objective norms* constructing the practices (“Technology Development as a Normative Practice” section). Passing through such foundational concerns, the main line of discussion of the paper is dedicated to proposing technology development activities to be reflected upon as normative practices. Dooyeweerd’s *Reformational Philosophy* will enable us to have a comprehensive view of various aspects of technology development, and suggest an overarching account to recognise and prioritize the different built-in values of such practices (“Case Study: Damming as a Normative Practice” section). Next, the study will concentrate on two considerable cases of ‘damming’: the inspiring case of Abbasi Dam, and the challenging case of Zayandeh Rood Dam. This field of engineering, as one of the most critical but controversial, multifaceted subjects of ethical concerns, will provide a rich background to illustrate the applicability of the account proposed in this study—particularly, in terms of contextual and historical views in considering value conflicts. Lastly, the paper will end with some general concluding points as well as recommendations for further research.

## Technology Development as a Systemic Multi-aspect Practice

A foundational critique regarding most current approaches to engineering ethics is ascribed to ‘black-box’ thinking which barely penetrates the intricate nature of technological development practices. That is to say, the complex processes and manifold aspects and features of technological developments scarcely come into analysis in such approaches; the focus is mainly on analysing the consequences from the outside (Pitt 2011; Van de Poel and Verbeek 2006).

In order to be able to address this concern, this study suggests that most technology development practices be understood first of all as multi-aspect systems—involving different peoples, institutions, companies, and infrastructural entities (Barkane and Ginters 2011; Geels 2002, 2004, 2005a, b; Geels and Kemp 2007; Musango and Brent 2011). Such typically ‘socio-technical systems’ have essential features, among which the following can be highlighted as relating to this research:

- They can have a complicated nature embracing numerous elements with an interwoven network of mutual relations, depending on various factors (Carlsson and Stankiewicz 1991; Geels 2002, 2004, 2005a, b; Georgieva 2008).
- Such systems, although adapted to particular economic, political, or other social characteristics in the development phase, are of considerable potential, particularly in the case of large technological systems (LTS), to lead to a consolidation phase in society—a *momentum* (Hughes 1969, 1994), “difficult to change, creating an appearance of autonomy from its environment” (van der Vleuten 2009, p. 219).
- They, in a level, construct socio-technical *regimes* comprising several *subsystems* of dynamic *actors* and *rules* (Geels 2004, 2005a, b; Geels and Schot 2007). The concept of *actor* in this account embraces a wide-ranging continuum of human actors and users, firms, industries, social groups, public authorities, research institutes, governmental organisations, etc., in a context of complicated interrelations and various features, perceptions, norms, and so forth. These *regimes* are consequently dominated by an extensive *subsystem* of subsequently different *rules*. These various rules do not have an independent nature and function; they are defined and work in strict relation to each other (Geels 2002, 2004, 2005a, b; Geels and Kemp 2007; Rip and Kemp 1998).

Therefore, such a wide-ranging perspective to the systemic nature of technology development practices undeniably calls for a concrete account capable of embracing the mentioned complexities of diverse aspects—including their various types of rules—in order to be able to recognise and prioritise the coexisting values of those systems.

## Technology Development as a Normative Practice

The ‘normative practice’ view has great potential to yield a concrete account to address most of the above-mentioned issues and deliberate and describe the complex nature of the technology development practices. The root of this perspective on ethics can be traced back to the critiques regarding the efficiency and applicability of the customary ‘predominant applied ethics’ (PAE), the inspiring critiques which conform to the brief content of the introduction of this study, as well.

The predominant applied ethics, in the view of scholars such as Jochemsen (2006), suffers from considerable challenges, namely:

- Concentrating on dilemmas, instead of referring to a broader view of a good life;
- Dealing mainly with the application of ready-made theories to overcoming the raised dilemmas, and crises, and regularisations and normalizing the theories, rather than tackling the probable ill-defined scientific and technological causes;
- Legitimizing the predominant developments;
- Ignoring the specific social contexts; and
- Rejecting the significance of worldviews in (ethical) debates

In quite a similar vein, Glas (2012) believes that such approaches to applied ethics—*principle*-*based* ethics, in his terms—“reduce moral deliberation to the application of general moral principles or rules to practical situations” (p. 4). For him, these predominant views have two crucial problematic weak-points: (1) they are too general to do justice to the particularities of intricate moral situations, particularly in highly technological contexts of practices, and, more importantly, (2) “by placing moral principles above or outside [a] practice, the impression was given that the moral dimension, instead of being a natural part of [that] practice, should be added from outside” (p. 4).

That said, the proposed ‘normative practice’ view benefits from a more concrete perspective in the view of the aforementioned scholars. As a *practice*-*based* approach (as opposed to the aforementioned *principle*-*based* one), this view is based upon a central tenet that

ethics is not just a special kind of decision-making skill to solve ethical dilemmas the practitioner is confronted with. … Ethical issues [rather] should be placed in the context of the integral normativity of the practice as can be formulated in all the constitutive principles and rules … whose realisation requires the related virtues of the practitioner (Jochemsen 2006, p. 107).

The normative practice view has already been applied to some subjects of study and could deliver outstanding insights to explain the normative aspect of the intended practices and present an organized manner of understanding the nature and state of different values within them (see, e.g., Glas 2012; Harandi et al. 2014; Hoogland and Jochemsen 2000; Jochemsen 2006, 2013; van Burken 2013; van Burken and de Vries 2012). In the same vein, this view is capable of explaining the nature and different features of values in technological practices.

‘Practice’ in such a view implies the meaning intended by McIntyre (1981) in his development of the theory of ‘social practices’ as:

[A]ny coherent and complex form of socially established co-operative human activity through which goods internal to that form of activity are realised in the course of trying to achieve those standards of excellence which are appropriate to, and partially definitive of, that form of activity, with the result that human powers to achieve excellence, and human conceptions of the ends and goods involved, are systematically extended (p. 175).

Through this definition, McIntyre has indeed attempted to present a meaningful, realistic description of humans’ (collective) actions in which certain ‘values’ are being realised (Verkerk et al. 2007), avoiding the trap of the *individualistic* and *liberal* ethics customary in most current approaches to analysing the state of different values embedded in practices (van Burken and de Vries 2012). For McIntyre, values have a meaning-based nature that relates to the ‘internal goods’ of practices, and, therefore, any ethical reflection on practices should be realised from the perspective of their inherent normativity, rather than being thought of as add-on components dependent on or constructed by outsider norms, rules, and obligations (van Burken and de Vries 2012).

Two key points must be explained at this point. First, the concept of ‘internal goods’ is different from so-called ‘goals’ typically set by and related to individual/collective actors. An internal good is the *destination* of a practice; the *finality* which belongs to the very nature of that practice or, in other words, the core value and reason appreciated within society and for which such a practice mainly exists (Jochemsen 2006, 2013; polder et al. 1997; Van Burken and Essens 2010); needless to say, a finality “leaves space for a number of subjective goals which could be set within [its] specific practice” (Polder et al. 1997, p. 414). For instance, the meaning of a medical practice, as evident for society, is ‘giving care’ which resides mainly in the good caregiving itself; it is not just determined by the measurable effects of the practice on patients’ health and also not to be thought of as the economic-oriented aims of the practitioners, such as profit or even earning a livelihood, although the practice may already lead to these results.

Secondly, the finality of a practice is realised well if a constellation of the ‘normative rules’ of that practice is simultaneously observed (Hoogland and Jochemsen 2000; Jochemsen 2015). That is to say, the competent performance of a practice is grounded in the ability to act according to the specific ‘rules’ that set up that particular practice and, at the same time, “define excellent practice and provide criteria to evaluate the activities of individual practitioners” (Jochemsen 2006, p. 104). One should notice that the concept of ‘rules’ in this view does not refer so much to the ‘knowing that’ types of rules, which have to do with the capability of articulating the applied rules in an explicit manner. Rather, it implies primarily the implicit side, that is, the ‘knowing how’ rules. As such, these ‘rules’ have intrinsic normative natures so that they can even be followed without a permanent conscious decision of the practitioner when applied. Thus, a competent practitioner must have certain virtues in order to be able to adeptly observe the related normative rules of practice and, consequently, to effectively fulfil the practice’s built-in finality. This is, indeed, the way in which the meaning-based virtues of a practice are realised (Jochemsen 2006, 2015).

McIntyre’s proposed account, nonetheless, is not by itself rich enough to describe the nature of such rules in more detail and to do justice to the complexity of practices. Needless to say, the values embodied in social practices are much more interwoven than explainable merely in terms of dualities such as internal/external and implicit/explicit norms (see, e.g., Verkerk et al. 2007; Van Burken and Essens 2010). And, as far as our intended practices—technology development practices—are concerned, there are two more significant points that must be taken into account.

**Point 1** Technology development is mostly an extensive practice entailing a coherent form of sub-practices; consequently, as a whole, it has a main finality, the realisation of which is based on harmonious performance of those different sub-practices and, subsequently, their various sub-finalities. The same holds regarding the embodied rules and virtues of the sub-practices, so that any significant tension or conflict among them (and their correspondent finalities) will not be likely to lead to a virtues, desirable result (see, e.g., van Burken 2013)

**Point 2** The normative rules of a practice, as indicated by scholars such as Jochemsen (2006) and van Burken (2013), can be conceived as its ‘rules of play’—formed based on specific normative principles, logic, and criteria. Hence, these rules are not very flexible to change or manipulation. They are typically shaped in the course of related ‘socially established human activities’ and can only be explained and realised from such a perspective to their particular features and co-relations. Dooyeweerd’s *Reformational Philosophy* can play a critical role in recognizing and understanding the different types of such rules of play in terms of the *constitutive* and the *regulative* normative rules, as described later.

The next subsection presents a broad explanation as to these two points.

### The Normative Structure of the ‘Rules of Play’

In order to be able to recognise and analyse the normative rules of playing in a technological practice, we would like to make use of Hoogland and Jochemsen’s (2000) approach, elaborated more in Jochemsen’s (2006). Drawing from Dooyeweerd’s ontological theory about the reality of things, their reflections propose that the essential ‘rules of play’ of a practice (respectively, the practices of ‘medicine’ and ‘nursing’) be defined and explained through distinguishing between a practice’s *constitutive* and *regulative* sides, the approach later extended to different specific practices such as ‘husbandry’ (Jochemsen 2013), ‘military service’ (van Burken 2013; van Burken and de Vries 2012), and ‘water management’ (Harandi et al. 2014).

In Dooyeweerd’s ontological account, the reality of things is subject to fifteen *spheres* (aspects) of meaning (properties) and laws. Things start to function actively from the first sphere, i.e., the *quantitative* one, and then go sequentially toward the next until it is finally qualified in a specific aspect, depending upon the nature of those things. For instance, as shown in Table 1, a rock is qualified *physically*, a tree is qualified *organically*, and an animal is qualified *psychically*. The concept of ‘things’ in this account also embraces all human practices; one can realise, for example, that the work of a company manager is typically qualified *economically* (see, for more detail, Clouser 2009). Furthermore, being qualified in each aspect means covering all previous spheres as well; for instance, the company manager’s professional activities also embody the *organic*, *lingual* and *social* spheres.

**Table 1:**
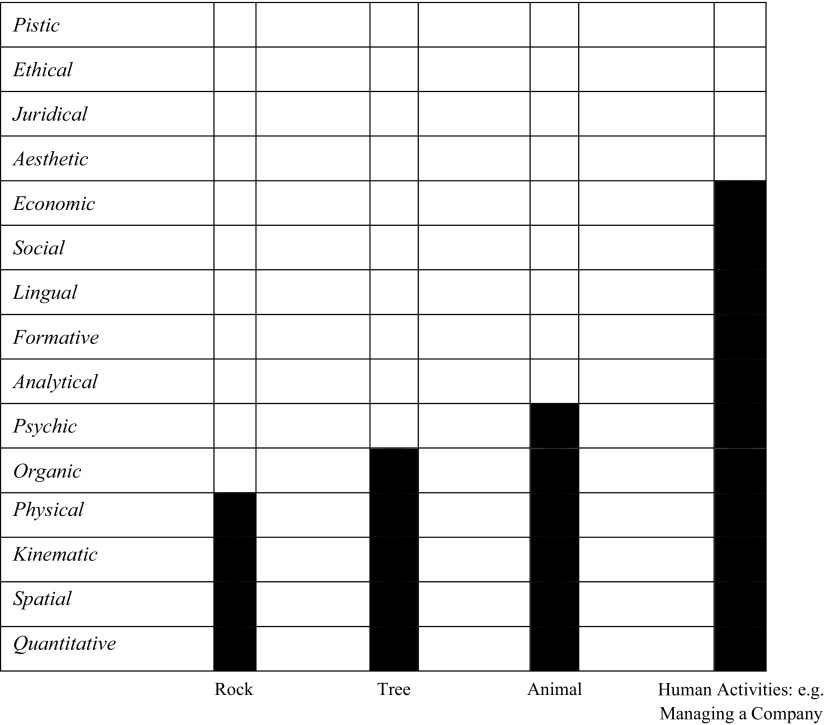
A schematic of things as qualified in Dooyeweerd’s view (taken with some improvements from Clouser 2009)

The *constitutive* side of a practice is defined as the side comprising the rules that ground the structure of that practice, in terms of the rules, processes, and (inter)actions that form that practice as it is. In other words, this side has to do with the constellation of principles and norms that characterize the social structure of the practice, what it aims for, and concurrently establishes its boundaries (Glas 2012; Hoogland and Jochemsen 2013; van Burken 2013; van Burken and de Vries 2012). The constitutive aspect can actually be conceived of as the ‘field of play’ of a practice and consists of three types of normative rules:

1. The *qualifying* rules which establish the finality (destination) of a practice and characterise it as it is. They are derived from the principle of the *qualifying* sphere of a specific practice.
2. The *founding* rules related to the fundamental activities that form a specific practice in the sense of its structure and content. They pertain exactly to the *formative* sphere of that practice.
3. The *conditioning* rules which formulate certain conditions of the context upon which a practice is performed, i.e., the rules of the *social*, *economic*, and *legal (Juridical)* spheres.
  (Hoogland and Jochemsen 2000; Jochemsen 2006)

To make this explanation clearer, it is worth drawing on Searle’s (1969) chess metaphor. The main aim of playing chess is ‘joy’—playing a game and having fun. Therefore, the matter of winning or losing is a secondary aim. Thus, playing chess is qualified by the ‘joy’ of its players, and, accordingly, its *qualifying* rules should be conceived as ‘those which lead to such joy’. Regarding the *founding* rules of this case, however, they have to do with the activities of playing chess, i.e., the typical technical rules of moving the pieces. Finally, the *conditioning* rules are the rules which set the context in which the game is played, namely, the specific rules of the structure of the board.

Turning to the description line, the *constitutive* rules (along with the *formative* and *constitutive* ones) need to be complemented through an interpretive side—i.e., *regulative* side. This side, also referred to as the ‘*directional’* side, pertains essentially to the attitudes, motives, beliefs, and the normative convictions that construct one’s worldview and shape one’s interpretive meaning-giving framework (Hoogland and Jochemsen 2000; Jochemsen 2015; Polder et al. 1997). So, there is no neutral performance of a practice in this view, and the *directional* rules play essential roles in this regard. They, although not immediately apparent to us, “function strongly in the form of unwritten, sometimes even unspoken codes of conduct and customs, convictions on what is decent and indecent” (Jochemsen 2015, p. 102).

Hence, in order to have a comprehensive image of a practice and make a concrete critique, one should take its *regulative* side, too, into explicit account; otherwise, one’s image of that practice can itself be subject to divergent and relativistic analyses and interpretations, as seen in the case study section. That said, the *regulative* side of a practice has mainly to do with the topmost sphere of its reality, that is, the *pistic* aspect, and many culture-based differences in viewing, analysing, and assessing a practice can be attributed to this aspect of normativity. Turning to the metaphor of chess, this side has to do with how one interprets the concept of ‘joy’ in such a game. That is to say, one may realize this ‘joy’ as either ‘(just) conquering’, ‘wining with a specific strategy’, ‘particularly-targeted practicing’, or even ‘simple, childish fun with children’.

Let us finalize this section by re-emphasizing the essential point that the competent performance of a practice requires the simultaneous realisation of all the above-mentioned normative rules.

## Case Study: Damming as a Normative Practice

Damming, as an essential component of water management systems, has undoubtedly been one of the most controversial subjects of ethical concern. Rooted in ancient human history going back more than 3000 years (Gourbesville 2008), dams have been frequent centrepieces of multi-aspect systems, involving numerous facets of both natural and socio-technical sides of ecosystems, and have led in many cases to multi-layered *seamless webs* of technological structures (as intended by Hughes 1986), particularly in the modern era (Adger et al. 2005; Molle 2007; Robbins 2004; Sneddon et al. 2002; Worster 1985).

That said, dams have been subject to different levels of achievements or failures in ethical terms (Molle 2006; Molle and Mamanpoush 2012); this makes their complex nature worth analysing in terms of their various normative features, particularly when taking into consideration the case that most of the failures in damming have brought catastrophes on their ecosystems (Molle 2006; Molle and Mamanpoush 2012). Two cases have therefore been selected to be studied in this section: the Abbasi dam as an ancient but successful case of sustainable development, and the Zayandeh Rud dam as a modern but unsuccessful one. Both cases have been selected from Iran, a historically water-based civilization which has embraced many types of water infrastructures in the course of history.

### A Brief Introduction of the Cases

**Fig. 1:**
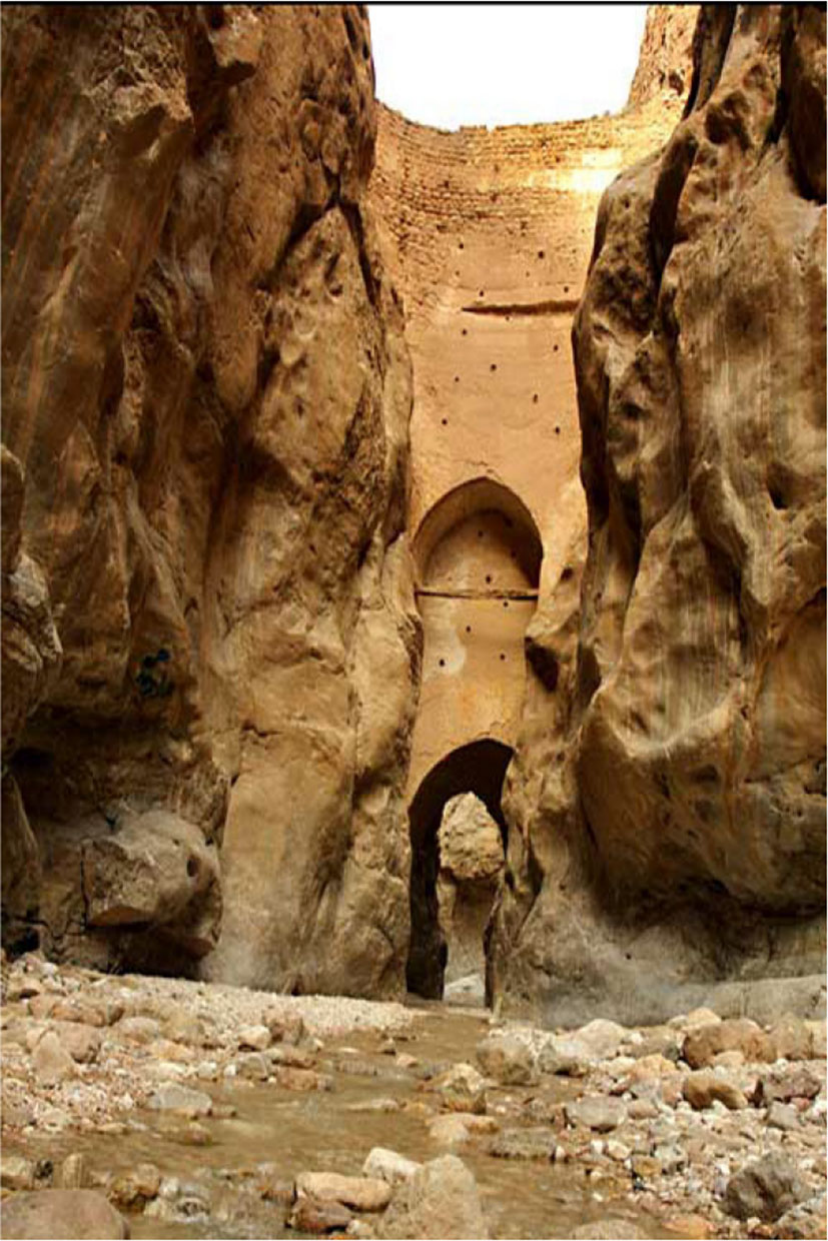
The Abbasi dam. Retrieved from http://www.kojaro.com/2016/7/17/120574/shah-abbasi-dam/

The Abbasi flood-retarding dam (Fig. 1) was constructed among the mountains near Tabas city and on the Nahrain River (one of the most important water resources in that region) in the east of Iran. Most historians identify the Safavids (1501–1722) as the origin of this dam. Built in a valley, it consists of two brick arches and a body made up of stone and mortar. Resting on the mountains on both sides of the river, the lower arch width narrows to 35.2 m in the lowest row. The distance between the top of the arch and the edge of the sharply pointed layer is 7 m. An interesting detail of this dam is the form of the bricks’ array, which is not limited to a certain width but is radially extended toward the mountains and has a uniquely strong structure. The dam is decorated with stone engravings of antelope, which are a symbol of the ‘appeal of water abundance’. The Abbasi dam has attractive features for tourists and in particular attracts nature enthusiasts throughout the year. With a height of 60 m, it is not only the oldest and the largest arch dam in the world but was also known as the tallest for 550 years. It has another distinction which no other dam can claim: the Abbasi dam’s 1 m-wide crest is still the thinnest in the world (Emami et al. 2005).

To get a more detailed look at its functional features, we quote from ‘Creative Harmony with Floodwaters by Value Engineering’ (Emami 2005):

The Abbasi flood-retarding dam is an illustrating example of water-oriented wisdom of the builders… The dam has protected the city of Tabas from floods of [the] Nahrain River for 600 years. To [avoid] construction of diversion tunnel[s], Iranian [builders] used to construct their dams on a brick arch in narrow canyons. The lower part of the dam was constructed during a dry season. This creative scheme has been used in many historical dams in Iran. At [the] Abbasi dam site, the lower part was not constructed so during floods the outflow from the dam was automatically regulated. The scheme is so elaborate that most of the engineers visiting the site believed that the dam was uncompleted or [that] it had suffered a wash-out because of the alluvium foundation… This is the first time that based on site visits by dam and flood experts and communications with the nearby villagers, the dam is called a flood-retarding dam. The dam site is located 100 m upstream of water springs that account for a considerable part of the base flow of the river. Consequently, it is unlikely that the main function of the dam is water storage, otherwise they should have constructed the dam downstream of the springs. A historical document indicates that the main function of the dam is controlling the floods.^2^

What qualifies this dam as an important civil technological building in the water resource management area is that, despite its great age, it is still stable and useful, and there has never been any indication that it has caused any problems, side effects, or environmentally harmful effects; it is, in fact, an exemplary case of sustainable development (Emami et al. 2005).

**Fig. 2:**
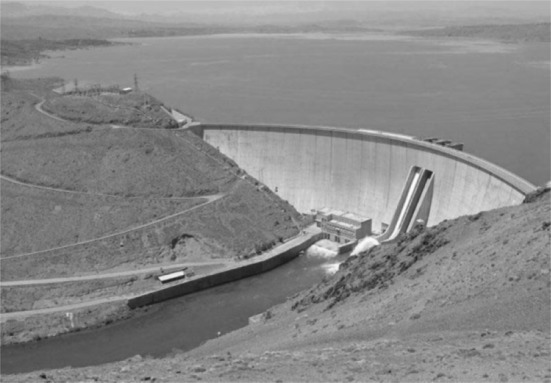
The Zayandeh Rud dam. Retrieved from https://www.tasnimnews.com/fa/news/1395/01/26/1048789/%D8%B3%D8%B1%D8%B1%DB%8C%D8%B2-%D8%B4%D8%AF%D9%86-%D8%B3%D8%AF-%D8%B2%D8%A7%DB%8C%D9%86%D8%AF%D9%87-%D8%B1%D9%88%D8%AF-%D8%B5%D8%AD%D8%AA-%D9%86%D8%AF%D8%A7%D8%B1%D8%AF-%D8%AD%D8%AC%D9%85-%D8%A2%D8%A8-%D8%B3%D8%AF-%D9%81%D9%82%D8%B7-260-%D9%85%DB%8C%D9%84%DB%8C%D9%88%D9%86%D9%85%D8%AA%D8%B1%D9%85%DA%A9%D8%B9%D8%A8-%D8%A7%D8%B3%D8%AA

The next case, the Zayandeh Rud dam (Fig. 2), is, however, completely different from the previous case: besides failing to accomplish its declared missions, this dam has also brought about many environmental problems. This case, indeed, has little relation to sustainable development in practice (see Adger et al. 2005; Molle and Mamanpoush 2012; Molle and Wester 2009; Morid 2003; Murray-Rust and Droogers 2004; Wilbanks 2006).

The dam’s name is taken from the river on which it is built—the Zayandeh Rud river, which means ‘the procreator river’ in Persian. This river, one of the largest rivers of Iran, is located in the Central Plateau of this country and, flowing from west to east, has been the lifeline of civilization in that extensive area. It irrigates and makes possible many gardens and farms along the way; it is the main source of verdure and fertility in the large, well known, and ancient city of Isfahan and its region (Molle and Mamanpoush 2012; Moradi 2014; Murray-Rust and Droogers 2004; Ranani 2014).

The dam was built in the early 1970s, 110 km west of Isfahan. This 2-arch dam has a 452 × 6-m crest, a height of 100 m, a maximum reservoir capacity of 1450 million m^3^, and a useful reservoir capacity of 1250 million m^3^. The maximum surface area of the lake behind the dam is about 54,000 m^2^. The dam was established with the proposition of providing benefits to different water users, with the following objectives:

- To irrigate the Isfahan fields;
- To increase areas dedicated to cultivation and to provide more sources of revenue;
- To supply the water demand of some regional industries;
- To protect the city and especially its ancient bridges against the Zayandeh Rud river flooding;
- To supply electricity to Isfahan.

**Fig. 3:**
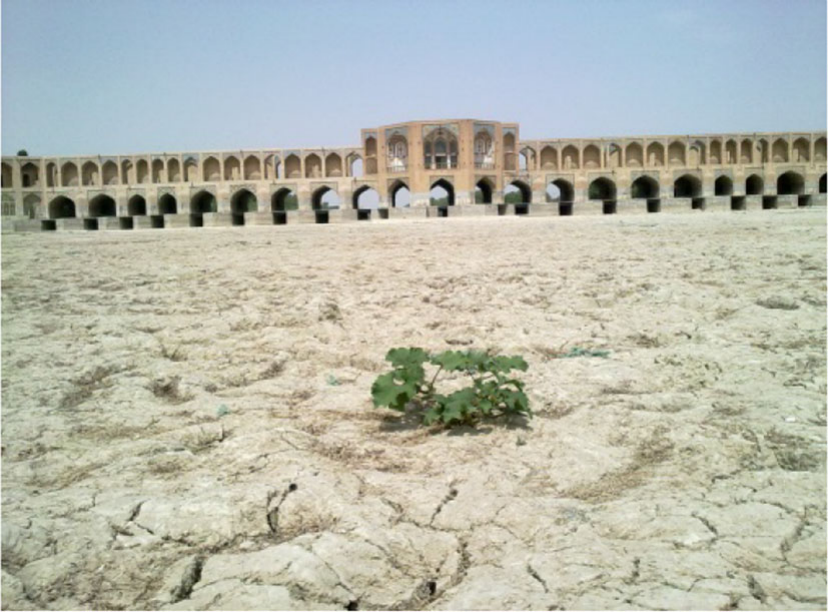
The Zayandeh Rud riverbed in the centre of Isfahan city is mostly dry. Retrieved from http://lastsecond.ir/news/Zayandeh-Rood

That said, despite the situation that in the past one could see a large amount of water in the bed of this river entering into the Isfahan region (Fig. 3), the main bed nowadays is almost dry, and the ‘procreator river’—once one of the most attractive and touristic settings of the region—is now suffering from various problems in its environment and the surrounding society, as elaborated below.

### Toward the Normative Practice View

The normative practice approach can describe the selected cases and analyse different reasons for their success or failure. As seen, pondering the constitutive and regulative rules of the practice of dam construction in general will pave an appropriate path forward.

(a) *Qualifying constitutive rules of damming* One may, first of all, find the topic of damming rather controversial or far from accessible. That is to say, the ideas addressing the primary ‘why’ behind the construction of dams appear considerably divergent in real practice: some may relate it to ‘increasing the economic growth of an area’ (and consequently of the country) and, on the other hand, some may emphasize subjects like ‘advancement of certain industry sectors’ as a purpose. ‘Gaining more political power or control’ can also be, not surprisingly, set as the main priority from the perspective of the political sector. Hence an essential question at this point is:

What is the actual qualifying sphere of damming?

One can draw on several definitions in this regard that extensively assert various reasons for damming. Poff and Hart (2002), for instance, begin their fascinating work *How dams vary and why it matters for the engineering science of dam removal* with the following paragraph:

Dams are structures designed by humans to capture water and modify the magnitude and timing of its movement downstream. The damming of streams and rivers has been integral to human population and technological innovation. Among other things, dams have reduced flood hazard and allowed humans to settle and farm productive alluvial soils on river floodplains; they have harnessed the power of moving water for commerce and industry; and they have created reservoirs to augment the supply of water during periods of drought (p. 659).

Another statement by Farhangi (2012) articulates an eloquent definition:

Dams offer security against two extremes: Against a lack of water bringing drought, power failures, dried out river beds and falling groundwater levels, and against too much water, especially too much too quickly, in the form of raging floods causing devastating inundation to farmland and people’s homes (p. 47).

Some key phrases can be highlighted in such statements: ‘to capture water and modify …’, ‘integral to human population’, ‘harnessing the power of water’, ‘allowed humans to settle’, ‘security against lack of water’, and ‘security against too much water’. These all can be said to be associated with a central concept regarding the *finality* of dam construction, that is, *welfare,* which belongs to the *juridical* aspect of Dooyeweerd’s proposed spheres of temporal reality (see Basden 2011). This being said, the normative rules of providing welfare should dominate all other objectives in this account.

(b) *Founding constitutive rules of damming* As previously discussed, this type of rule has to do with the *formative* sphere, in this case, all necessary techniques and skills of damming. Therefore, the rules essential to and used in planning, designing, constructing, assessing, maintaining, managing, and the like, whether written or unwritten, are the binding rules that can be described as the normative *founding* rules.

(c) *Conditioning constitutive rules of damming* These are the normative rules that dominate the context in which the damming practice takes place and can be of a local as well as global nature. The rules associated with matters such as environmental circumstances, official rules, governmental support, legal background, and so forth, as far as dam construction is concerned, belong to this kind of normative rule, which can be related to the *physical*, *social*, *economic*, and *juridical* aspects.

(d) *Regulative rules of damming* These rules, as discussed earlier on, are concerned with concepts such as ‘attitudes’, ‘beliefs’, and ‘motives’ behind the practice of damming; the rules which lead to an exclusive interpretation of the practice’s *constitutive* rules, particularly concerning the *finality*—i.e., the *welfare*—of this practice. From this perspective, one can raise the following questions regarding how to view and interpret the concept of *welfare*:

- What is meant exactly by this welfare? How can different types of welfare, e.g., ‘economic welfare’ and ‘safety welfare’, be identified and distinguished from each other in this case?
- Whose welfare matters in damming? How should different people, particularly those on the upstream and downstream sides, be considered in terms of benefit from such welfare? To what extent and according to which criteria is the welfare of some people allowed to be sacrificed for that of others?
- Is this welfare defined only in the sense of human welfare, or does it include the environment’s welfare as well? To be more specific, how should the concept of ‘sustainable development’ be considered in this sense?

From Dooyeweerd’s perspective, the *regulative* rules of damming, although having to do with the *lingual* aspect (when seen as explicit interpretations), mainly belong to the *pistic* spheres; these types of rules primarily arise from one’s view of the world and the meaning of welfare in one’s point of view (see Basden 2011; Dooyeweerd 1955).

Now let us take a more detailed look at the intended cases in the sense of how they satisfy their inherent normative rules.

### The Abbasi Dam and Its Normative Rules

Applying the normative practice approach to the Abbasi dam yields a worthwhile insight as to how it can be considered an exemplary case of sustainable development. To begin with, it is worth taking a look at its *qualifying rule*—the *norm* of providing *welfare*—and interpret it through the *regulative* side: The main function of this dam was protecting the people against the seasonal floods of the Nahrain River, which was prone to become a terrible torrent threatening the local inhabitants’ life. The dam was therefore built in such a way that it could control a natural disaster but without any serious conflict with its environment: its special shape leaves the torrents not entirely blocked, but controlled and retarded.

Furthermore, one can see that the other normative sub-practices (each with their own qualifying rules), have also been taken into account in designing and developing this dam: for instance, the role of communication and traveling through the riverbed for the people of the surrounding villages has not been affected, and the equitable distribution system of the water of Nahrain Qanat has been preserved (Emami 2005, 2014; Emami et al. 2005). These all, as discussed earlier on, pertain to *conditioning* rules that seem well considered in this case.

It is moreover worth mentioning another well-considered instance of *conditioning* rules, which has to do with the historical background of this context, that is, the role of *Mirabs* (plural of *Mirab*) in the water management system dominant in that zone. Mirabs were locally well-known and trusted people who had the responsibility of managing particular parts of a water system. Each Mirab, usually born in that particular region, was a knowledgeable authority concerning water system issues as well as the various features of the lifestyle of people in his region. This Mirab-based system had been defined based on a systematically and gradually constructed mechanism over the course of many years, which led the Mirabs to play a significant role in making essential decisions as to renewing or modifying their related water management systems (see, for more details about Mirabs, Balali et al. 2011; Harandi et al. 2014; Hossaini 2006; Mehraby 2010; Mohmand 2011; Molle and Mamanpoush 2012; Thomas and Ahmad 2009).

The *founding* rules of this case, also, can be seen to be dependent on the status of the Mirabs and are defined and flow well in such a relation: the ‘managerial’ side of the necessary knowledge and skills, certainly including the tacit ones, is directly formed and run through the Mirabs’ direct influence, and the purely technical side as well (i.e., the rules concerned with the process of designing and constructing the dam) come into being within such a supervisory chain.

**Fig. 4:**
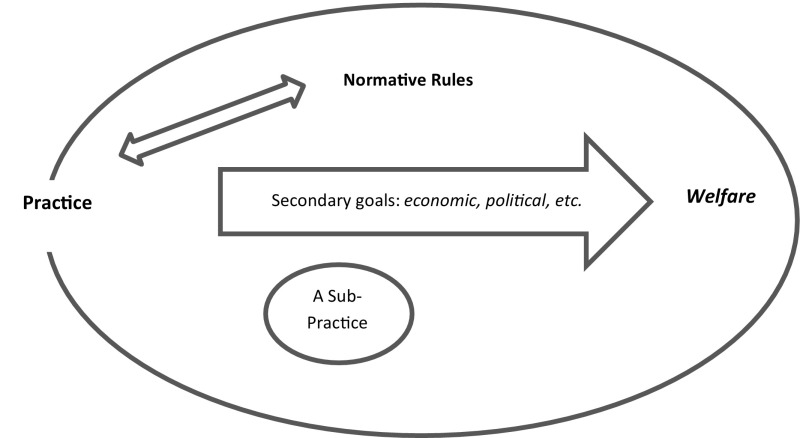
A normative practice as it applies to for the Abbasi dam (taken with some changes from Jochemsen 2013)

To recapitulate this case, one can observe that the intended *welfare* is attained through simultaneous adherence to its constellation of normative rules, so that one does not disturb (pre)established practices (Fig. 4); this has led the dam to become a sustainable development, as concerns both the environment and human nature: “[it] is a dam for all generations and no one could imagine any limitation to its useful life, unless it were sacrificed in human development programs and drowned in the reservoir of a new dam” (Emami et al. 2005).

### The Zayandeh Rud Dam and Its Normative Rules

The Zayandeh Rud dam (along with its river and basin) as a modern technological structure^3^ has been subject to many challenges and has raised numerous problems in relation to the surrounding nature and human life (Khatounabadi 2014). This extensive, multi-layered, multi-sectoral, multi-causal, and complex system of numerous variables (Biswas 2004) has been organized in such a way that it could not satisfy its intended major missions and has conversely brought about considerable difficulties for different types of regular users—the downstream users in particular—as well as the environment, not to mention future generations (Harandi 2016; Molle and Mamanpoush 2012). The troubles of this dam are mainly attributed to an inadequate understanding of all the interacting sub-systems of such a complex infrastructure. From the development’s inception, this confusion has led to significant consolidated conflicts and regular tensions over matters such as river rights, involving actors such as the state, farmers, factories, and others (see, for more detail, Harandi 2016; Molle and Mamanpoush 2012; Molle and Wester 2009; Molle et al. 2009). These tensions appear unresolvable through customary technical or managerial approaches, or through simply engaging different stakeholders of various backgrounds, aims, and even immutable views and values in dialogue and closer cooperation (Harandi 2016). However, analysing the case through examining its normative rules will yield insights about the case from the perspective of what it *ought* to be, as compared to what it currently *is*.

Let us begin this with considering the *welfare* (the *qualifying* norm) in this case, through taking its probable interpretations (*regulative* norms) into account. The main problem seems rooted here and is reflected in inconsistent approaches to realising the multi-purposed set of intentions regarding this dam. That is to say, the chief *welfare* in this case, as a social normative practice, should have been defined in the sense of *appropriateness and due for all* (Basden 2011), serving not just *me* and *mine*, but *we*, *us* and *them*, indeed the *whole,* including nature. Unfortunately, this finality has been violated and sacrificed at the expense of certain sectoral goals.

**Fig. 5:**
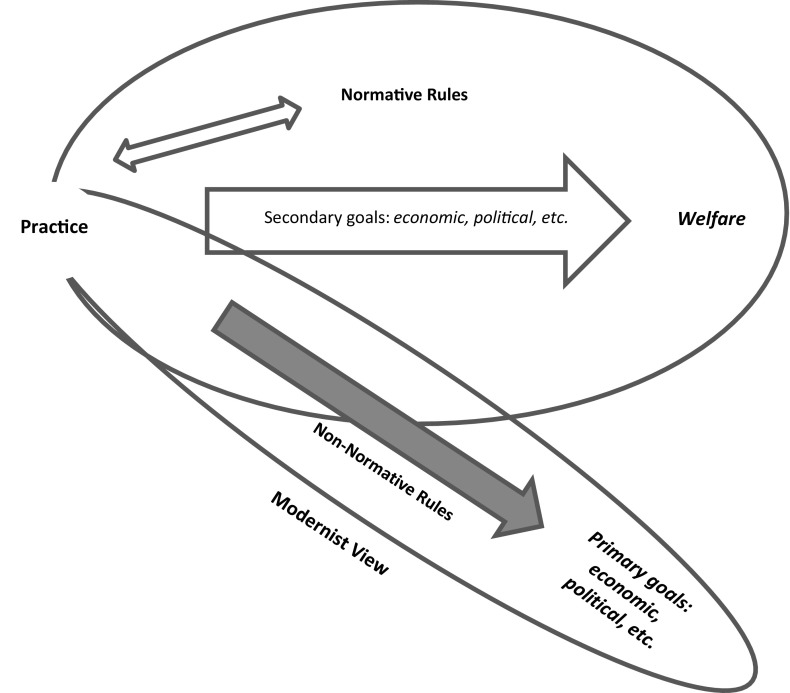
The Zayandeh Rud dam has violated the normative rules

The sectoral goals defined in the course of establishing this dam included a ‘joint electricity-irrigation scheme’ (Harandi 2016). Besides ‘protecting the people of the Isfahan region against floods or droughts’, the dam was meant to ‘provide electricity for both people and industries’, ‘found a well-irrigating system for farming’, ‘supply drinking water, as well as water needed by the steel factories, refinery, and power plants in the area’, ‘transfer the water to the other zones’, and so forth (Harandi 2016). These goals, however, are qualified at quite different levels of reality. For instance, while the practices of ‘protecting people against floods and droughts’ and ‘providing drinking water’ are qualified *juridically*, the practices of factories and their owner companies are typically qualified in an *economic* sphere, as is the practice of farming. Nevertheless, the problem that has arisen here is that most of the mentioned goals have not been articulated in a well-ordered manner consistent with the finality of *welfare* in the sense of ‘due for all justly’ (Basden 2011); that is to say, one can observe numerous cases of prioritizing the economic (or political) goals of companies and the state above the normative *welfare* of the case with respect to people and the environment (Fig. 5). This has led to frequent droughts in the riverbed and consequently, many problems for the environment and the society of the region (Khatounabadi 2014; Ziaei 2014a).

A just welfare founds its core normative rules in relation to those of the *founding* and *conditioning* and therefore needs to be examined from this view also—recalling the significance of the simultaneous realisation of the entire constellation of normative rules. This consideration reveals some further aspects of the difficulties mentioned in the case that can be elaborated according to Fig. 5.

The construction of the dam with its multi-sectoral goals has failed to conform to its *conditioning* norms, particularly those belonging to the Zayandeh Rud river. This river, tied to a historical context of sociocultural norms, used to be the main source of verdure and fertility of the Isfahan region and played a critical role in supporting the farming (sub-practice) of the people of that region, as well as other traditional livelihoods (Harandi 2016; Khatounabadi 2014). The use of this water in this particular sociocultural context was long governed through the wisely structured system overseen by a Mirab, as elaborated by Molle and Mamanpoush (2012):

The river was managed by a Mirab and six assistants selected by 33 boluk representatives, with the help of appointed maadi salars, heads of each of the main run-of-the-river diversion canals (maadi) that were branching off the river. These managers were paid by users, proportionally to the amount of water received, and were dispensed with if their service was judged to be unsatisfactory (p. 287).

A Mirab must also “prevent the powerful from trespassing on the weak with regard to the shares of water” (Spooner 1974, p. 151), and “referee water disputes with the confirmation and approval of the local leaders” (Molle and Mamanpoush 2012, p. 287). This allowed the governance of this river system to be placed entirely in the hands of local people; it was a democratic governance in which the state-related governors rarely had a direct role (Hossaini 2006).

It is worth mentioning that the Mirabs’ managerial practice also followed the Civil Code of Islamic Law. This code played a significant role in establishing a just norm for water rights:

The Civil Code … gives priority to established owners of land over newcomers and upstream over downstream users of water. Prior appropriation rights [as well] were protected by a clause stipulating that the use of water by newcomers should not impact on the interest of existing users (Molle and Mamanpoush 2012, p. 291).

Some of these norms were locally regulated and “governed to a large degree the access to, and use of, water in irrigation within what was a complex organization of supply in an uncertain physical environment” (McLachlan 1988, p. 71). However, the case of the Zayandeh Rud dam was subject to many top-down policy and governance rules from the very first stages of its design and construction. This was in line with the modern view of the Iranian state regarding governance of water resources through a centrally integrated, government-based system. This view emerged in the field of water management with the Land Reforms of the 1970s and pushed aside many local traditions and norms which had evolved over the course of many decades and even centuries (Harandi et al. 2014).

The *founding* rules—the *formative* norms—of the described system had long been linked to these traditional norms and background, in terms of both technical and managerial competencies. For instance, one can point to one of the most essential policy documents governing such norms, *Sheikh*-*Bahai’s edict (Tumar)*. Sheikh-Bahai, a renowned Iranian scientist from the Safavid era, developed^4^ a comprehensive set of rules known as the *Tumar* that wisely considered many necessary guidelines and particularly local norms—including geographical, social, and technical ones—to be applied to both the design and governance of water management systems, along with the general (not regionally specific) knowledge and skills necessary for each of those domains. One can see, however, that the *Tumar* and much of the other local knowledge and skills pertaining to designing, constructing, maintaining, and managing such water infrastructures has been ignored in devising and establishing the Zayandeh Rud dam (see, for more details in this respect, Harandi 2016; Hossaini 2006; Molle and Mamanpoush 2012; Moradi 2014; Nasr ‘e Esfahani 2014; Ziaei 2014b).

In summary, the Zayandeh Rud dam can be understood as a case of inconsistent and conflicting norms. Its internal mission is not satisfied, but violated, through its existing form of governance. The next section will propose further suggestions for tackling these issues.

### The Normative Practice View: Toward Tackling the Case of Failure

The Zayandeh Rud dam has been subject to many conflicts in the course of its management and use, stemming, as already discussed, from its intricate engagement with diverse actors and stakeholders having different expectations and desires. ‘Negotiating’ over the conflicts seems to be an ineffective, abstract solution to the problem; in negotiations, the parties seek to regulate a win-lose discursive framework to maximise their own benefits from the existing limited water (Harandi 2016). Some concrete criteria are necessary to make the problems clearer and delineate the governing rules and their conflicts. Such a framework is needed to manage the competing sectoral perspectives and the anticipations of each stakeholder. Otherwise, the more interests that are represented, the more issues will arise and the more complexity must be overcome (Coughlin 1999; Harandi 2016).

Taking the normativity of technology development practices into account could provide practical insight into how to tackle or prevent the failures of the Zayandeh Rud dam case or similar cases. The problem has its roots, as mentioned earlier, first of all in diverse views on the aim of establishing the dam. That is to say, while the welfare of the people and protecting the environment should be considered to be its ultimate *finality*, this mission has been sacrificed to the economic gain of some powerful stakeholders, such as industries, or to political aims to convey the river water to particular planned developments, with the intention of greening the surrounding deserts, electrifying the country, spreading irrigation projects, and increasing agricultural outputs (Bouguerra 2006; Harandi et al. 2014; Molle and Mamanpoush 2012).

This problem, it should be noted, is attributed to the *modernist* interpretation of welfare, a utopian dream of subduing nature and mastering water for human prosperity, which emerged strongly beginning in the mid-nineteenth century (Bouguerra 2006; Harandi et al. 2014; Molle 2006; Molle et al. 2009). One can see this view manifested in many approaches to dams or their river basins. Nehru, when commissioning the massive Nagarjuna Sagar dam, spoke of dams as the ‘modern temples of India’. The Orange River Project was heralded as changing the desert face of South Africa into a paradise. Churchill stated that rivers should perish gloriously without a single drop of them reaching the sea. The dominant view of the beginning of the twentieth century in Spain was based on the Spanish motto that not a single drop of water should reach the Ocean without paying its obligatory tribute to the earth to make the country rich. Fidel Castro emphasized that not a single stream or river should be left undammed. Zemin (the president of China) related the Three Gorges dam of the Yangtze river to the daring vision of Chinese people for a new horizon and better future during their reform era, and so forth (Molle 2006).

These modernist views of dams and other water management projects did not set out to isolate such infrastructures from nature but gradually led to the dominance of regulative norms which did not qualify welfare in the juridical sphere of “due for all, far beyond me and mine, beyond ‘we, us, and them’ within our ken, to the whole” (Basden 2011, p. 18). It downgraded such qualifying norms to satisfy the tenets of productivism and utilitarianism and consequently, replaced the respect for nature and society as a whole with that for the sectoral demands typically belonging to the government and industry (see, for more detail, Harandi et al. 2014; Molle et al. 2009).

The modern approach to governing hydrological systems, founded in Iran’s 1968 Land Reforms, influenced the dominance of both the *conditioning* and *founding* normative rules as well and it swept them away in a dramatic manner. The normative rules of devising and managing water systems on the basis of local resources and indigenous knowledge and “implemented according to precise technical- and societal-tuned mechanisms” were substituted by the centrally-oriented regulations of the hierarchical governance systems of the state or state-based institutions (see, e.g., in Harandi et al. 2014; this is how the conventional Mirabs were replaced by the Mirab Company in the Isfahan region.) The problematical issue is that the new water management systems—particularly the new hydropower dams—are (components of) massive ‘distributed systems’ which, as typical post-modern technologies, embrace a diversity of conflicting technical, sociocultural, or environmental interests and contingencies. For that reason, from a ‘system view’, such massive distributed systems escape central control—not only technically, but also socially and politically. This makes it difficult for actors to appropriate the benefits of their interventions and to influence technological developments in the ‘right’ direction (Georgieva 2008; Rip and Groen 2001), and, hence, “the idea of a single institution (e.g., the government) that controls the entire process of technological development becomes a myth” (Georgieva 2008, p. 112).

We would like to end the analysis by making two complementary points:

First, although the discussion is based on delineating the normative rules of technology development, the analysis of the failed case attributed its problems mainly to its underlying modern thinking. This is not a surprising finding, and the reason lies in that the normative practice view is primarily based upon McIntyre’s account of the concept of practice, where he explicitly introduces himself as an ethicist against the current modern ethos (McIntyre 1981). Needless to say, the proposed approach to realising the normative rules is also a ‘meaning-based ethics’ product of Dooyeweerd’s ontology, believing that ‘meaning precedes existence’ (see, for more detail, Jochemsen 2006; Clouser 2009).

Secondly, one might raise a doubt about the applicability of the above analysis to tackling failures such as the Zayandeh Rud dam, in the sense that amending the dominant rules may not be entirely possible, or that further analyses in this line may lead to the conclusion that there is no possibility for amelioration of the case, and removing the dam may be the only option. We concur with this concern, that is to say, it is not always possible to solve such huge problems in their entirety and, in these cases, we may need to resort to either solving them partially or at least preventing them from being extended. The latter option was recently highlighted in some European water management policies. It must also be borne in mind that dam removal has recently been undergoing further study and has been put into practice, although this is not feasible in many cases (Lindloff 2000; Poff and Hart 2002). That said, what is intended in the philosophical descriptions is to illuminate or discover some new aspects or provide a concrete base of argumentation to perceive the subjects of study. In addition, damming is merely one case among many technological development cases, and the Zayandeh Rud dam is merely one case among many dams. The normative practice view is a holistic perspective to understand the inherent normativity of technology development as a whole and can be applied to different cases in various fields which may be much easier to tackle.

## Concluding Remarks

The normative practice approach to understanding different aspects of the practice of technology development can lead to valuable insights regarding various values within the complicated and multi-layered systemic essence of engineering activities. This view, in other words, can contribute to productive discussions about the controversial subject of prioritizing ethical and moral issues in engineering practice, discussions that used to be considered a blind alley in the view of some scholars of ethics and technology.

The normative practice view of technological development extends the area of reflections upon normativity of technology to its *volitional* aspect. These reflections were typically confined to the realm of epistemological perspectives on the nature of technology, related to understanding different features of technological knowledge (see, e.g., de Vries 2003, 2005, 2010; de Vries and Meijers 2013; Meijers and Kroes 2013; Sarlemijn 1993), or the analyses examined some partial aspects of technology, such as the character of technological ‘artefacts’ (Franssen 2009; Vaesen 2013), ‘risks’ (Möller 2013; Peterson and Espinoza 2013), ‘environmental considerations’ (Sandin 2013), ‘processes and functions’ (Radder 2009), and etc.

The proposed normative practice view, which stemmed from a meaning-based worldview, considers the values of a practice as built-in components of its coherent nature, the normative rules (standards of excellence) to be understood and considered in the course of related engineering activities, in order to achieve the intended finality of that practice. The qualifying, founding, and conditioning normative rules constitute a practice and define a significant part of its concrete form and the regulative rules that direct that practice and give a specific meaning to its intended finality. The approach as a whole can be considered as a perspective less likely to be captured in the trap of relativistic ideas and judgements regarding the moral and ethical issues in the course of technological developments. Applying such a perspective to the case of damming, as one of the most controversial fields of serious concerns regarding technological developments, could yield insights as to the matter of success or failure in such cases, as related to ethical issues. These issues are not easily solvable, due to the profound impact of the modern view and its accompanying enormous technological momentum.

## Footnotes

1 *Incommensurability* happens when “two or more values are incommensurable if they cannot be expressed or measured on a common scale or in terms of a common value measure” (Van de Poel 2009, p. 977).

2 As a flood occurs, [the] reservoir fills and the discharge increases until the flood has passed and the inflow has become equal to the outflow. After this time, water is automatically withdrawn from the reservoir until the stored water is completely discharged (http://www.tabasenc.ir/abbasi-dam-creative-harmony-with-floodwaters-by-value-engineering/).

3 Completed and came to use in 1970 https://fa.wikipedia.org/wiki/%D8%B3%D8%AF_%D8%B2%D8%A7%DB%8C%D9%86%D8%AF%D9%87%E2%80%8C%D8%B1%D9%88%D8%AF.

4 Jelveh Nejad (2014) believes that the *Tumar* was developed before Sheikh-Bahai and only improved or approved by him.
